# Solar UV Doses of Young Americans and Vitamin D_3_ Production

**DOI:** 10.1289/ehp.1003195

**Published:** 2011-08-18

**Authors:** Dianne Eyvonn Godar, Stanley James Pope, William Burgess Grant, Michael Francis Holick

**Affiliations:** 1 U.S. Food and Drug Administration, Center for Devices and Radiological Health, Rockville, Maryland, USA; 2 Sun Systems and Service, Inc., Oak Park, Michigan, USA; 3 Sunlight, Nutrition and Health Research Center, San Francisco, California, USA; 4 Boston University School of Medicine, Boston, Massachusetts, USA

**Keywords:** cancer, environment, sunlight, sunscreen, vitamin D

## Abstract

Background: Sunlight contains ultraviolet B (UVB) radiation (290–315 nm) that affects human health in both detrimental (skin cancers) and beneficial (vitamin D_3_) ways. Serum 25-hydroxyvitamin D concentrations from young Americans (≤ 19 years) show that many have deficient (< 50 nmol/L, 20 ng/mL) or insufficient (< 75 nmol/L, 30 ng/mL) vitamin D levels, indicating that they are not getting enough sun exposure. Those findings are in conflict with some calculated, published values that suggest people make “ample” vitamin D_3_ (~ 1,000 IU/day) from their “casual,” or everyday, outdoor exposures even if they diligently use sunscreens with sun protection factor (SPF) 15.

Objective: We estimated how much vitamin D_3_ young Americans (*n* = ~ 2,000) produce from their everyday outdoor ultraviolet doses in the North (45°N) and South (35°N) each season of the year with and without vacationing.

Methods: For these vitamin D_3_ calculations, we used geometric conversion factors that change planar to whole-body doses, which previous calculations did not incorporate.

Results: Our estimates suggest that American children may not be getting adequate outdoor UVB exposures to satisfy their vitamin D_3_ needs all year, except some Caucasians during the summer if they do not diligently wear sunscreens except during beach vacations.

Conclusion: These estimates suggest that most American children may not be going outside enough to meet their minimal (~ 600 IU/day) or optimal (≥ 1,200 IU/day) vitamin D requirements.

Ultraviolet (UV) radiation (290–400 nm) in sunlight can affect people’s health in both detrimental and beneficial ways. Short-term detrimental health effects include sunburn and immune suppression, and long-term detrimental health effects include cataracts, photoaging, and DNA damage with mutations that can lead to nonmelanoma and melanoma skin cancers. Beneficial health effects of UV radiation include medical detection and treatments of diseases such as cancers; possible reduction in the mortality from some cancers, including colon cancer (Garland et al. 1989), breast and prostate cancer (Freedman et al. 2002), and melanoma (Berwick et al. 2005); and vitamin D_3_ production (Holick et al. 1980).

Children may need more vitamin D_3_ than amounts recently recommended (600 IU/day) by the Institute of Medicine (2011) to maintain healthy muscles (Pfeifer et al. 2002), bones (Holick 2007), and general health (Heaney and Holick 2011; Holick 2011). For example, the risk of type 1 diabetes may be significantly reduced in children if they take 2,000 IU/day of vitamin D supplements early in life (Ponsonby et al. 2009; Zipitis and Akobeng 2008). Conversely, there is some evidence that infants who develop rickets, a consequence of vitamin D deficiency, are at increased risk of type 1 diabetes mellitus (Hyppönen et al. 2001). Thus, correct levels of vitamin D may prevent or reduce the occurrence of type 1 diabetes mellitus (Harris 2005).

For most children, the major source of vitamin D_3_ comes from exposing their skin to sunlight (Glerup et al. 2000). In animals, yeast, fungi, and plants, sunlight forms either vitamin D_3_ or D_2_, both of which may be equally effective in maintaining human serum levels of 25-hydroxyvitamin D [25(OH)D; D represents both D_2_ and D_3_; Holick et al. 2008]. Vitamin D_3_ production occurs in human skin when ultraviolet B (UVB; 290–315 nm) photons convert 7-dehydrocholesterol, or provitamin D_3_, made by keratinocytes to previtamin D_3_ (MacLaughlin et al. 1982), which thermally isomerizes to vitamin D_3_ (Holick et al. 1995b). Vitamin D_3_ that forms in the skin and vitamin D_2_ or D_3_ from dietary sources are carried into the bloodstream by vitamin D–binding protein, an alpha_1_ globulin. The liver enzyme vitamin D-25-hydroxylase hydroxylates it to 25(OH)D, and then the kidney enzyme 25(OH)D-1-α-hydroxylase further hydroxylates it to the hormonally active form, 1,25-dihydroxyvitamin D [1,25(OH)_2_D]. In addition to liver and kidney cells, most cells in the body can convert either vitamin D or 25(OH)D to 1,25(OH)_2_D, including colon, breast, lung, and prostate cells, keratinocytes, and melanoma cells (Reichrath et al. 2007; Zehnder et al. 2001).

Most people in the United States do not get sufficient vitamin D from dietary sources [fortified foods and drinks (milk and orange juice) and supplements] (Moore et al. 2004), so sunlight-derived vitamin D is their primary source. However, because the incidence of skin cancers is increasing at an alarming rate, public health organizations have warned people, especially children, to stay out of the sunlight whenever possible and to wear protective clothing, sunglasses, and sunscreens with sun protection factor (SPF) 15 or higher while outdoors from 1000 hours to 1600 hours. (e.g., Goldman 2002; Skin Cancer Foundation 2011; U.S. Environmental Protection Agency 2011). Although sunscreens with SPF ≥ 15 almost completely inhibit vitamin D_3_ production (Holick et al. 1995a; Matsuoka et al. 1988), the American Academy of Dermatology has concluded that people will still make “ample” vitamin D_3_ (≥ 1,000 IU/day) because they get plenty of “casual,” or everyday, outdoor UV exposure (Lim et al. 2005). However, an evaluation of serum 25(OH)D revealed that about half of all American children have either deficient or insufficient levels (Gordon et al. 2004, 2008; Looker et al. 2002, 2008; Mansbach et al. 2009).

To clarify whether “casual” sunlight exposures make ample vitamin D_3_, we calculated the amounts produced from everyday outdoor UV dose estimates (Godar 2001) according to sex, age, Fitzpatrick skin type (Fitzpatrick 1988), clothing (Matsuoka et al. 1992), and season for children in the northern and southern United States.

## Materials and Methods

We extracted and calculated erythemally weighted UV doses (Godar et al. 2001) from a 2-year survey of 9,386 Americans residing in the contiguous United States, including about 2,000 children (≤ 19 years) (Godar 2001). We converted the average daily standard erythemal dose (SED; a UV dose weighted by the erythemal action spectrum, so that it is independent of the spectral output of the source and the individual’s skin type; 1 SED = 100 J/m^2^) for each season to standard vitamin D_3_ doses (SVDs) relative to the horizontal plane using action spectrum conversion factors (ASCFs; Pope et al. 2008), and then converted those planar dose estimates to human body doses using geometric conversion factors (GCFs; Pope and Godar 2010). An action spectrum shows the relative effectiveness of each wavelength (nanometers) toward some end point that can be used to weight spectral outputs of different sources like the sun or a tanning bed in order to estimate amounts produced—for example, erythemal response (sunburn) or vitamin D_3_ production.

The ASCFs account for the differences between wavelength contributions estimated by the erythemal action spectrum and the previtamin D action spectrum toward previtamin D_3_ production. To derive the SVD for a given season and latitude, we multiplied the SED per day by the appropriate ASCF:

SVD = SED/day × ASCF. [1]

ASCFs for the northern (45°N) and southern (35°N) United States are 1.034 and 1.104 for summer, 0.879 and 1.029 for fall, 0.565 and 0.842 for winter, and 0.9 and 1.049 for spring, respectively (Pope et al. 2008).

SVDs, which represent horizontal plane or planar doses, are converted to whole-body doses using GCFs based on a full-cylinder model representing the human body (Pope and Godar 2010). GCFs for the northern United States (45°N) are 0.434 during the summer and spring and 0.508 during the winter and fall; GCFs for the southern United States (35°N) are 0.417 during the summer and spring and 0.484 during the winter and fall. The average vitamin D_3_ dose (VDD) per day is derived by multiplying the SVD by the appropriate GCF:

VDD = SVD × GCF. [2]

To estimate the amount of vitamin D_3_ a person makes from outdoor UV exposures when engaged in different activities, we first determined how much vitamin D_3_ a person would make from an erythemally weighted UV dose with uniform geometry, such as the UV dose from a tanning bed. For example, a female with Fitzpatrick skin type II (Caucasian; Fitzpatrick 1988) with a whole-body exposure to one minimum erythemal dose (MED), or the amount of UV needed to barely turn skin pink after 24 hr, in a tanning bed with a weighted spectral distribution similar to the midday summer sun at approximately 35°N produces the equivalent of an oral dose of approximately 15,500 IU vitamin D_2_ or D_3_ (Holick et al. 2008). Because melanin impedes the penetration of UVB and reduces vitamin D_3_ production (Clemens et al. 1982; Matsuoka et al. 1991), the UV dose required to achieve an MED and make the same amount of vitamin D varies by skin type. For Fitzpatrick skin type II (Fitzpatrick 1988), 1 MED is defined as 250–350 J/m^2^, and so we refer to skin type II as 300 J/m^2^ or 3 SED. For a whole-body exposure, a person with skin type II with an MED of 320 J/m^2^ will make approximately 15,500 IU/MED or approximately 4,900 IU/SED. Similarly, 1 MED is 300–500 J/m^2^ or 4 SED (average) for skin type III (olive skin tone, Hispanic or Asian), 450–600 J/m^2^ or 5.25 SED for skin type IV (brown skin tone, e.g., Indian), 600–900 J/m^2^ or 7.5 SED for skin type V (light-skinned African American), and for skin type VI (dark-skinned African American), 600–2,000 J/m^2^ or approximately 13 SED. Consequently, for a given UVB dose, Caucasians with skin type II make at least two to three times more vitamin D_3_ than do African Americans with skin type V (Dong et al. 2010; Matsuoka et al. 1991) and 10–20 times more than do African Americans with skin type VI (Clemens et al. 1982). The ratio of vitamin D production for someone with skin type II compared with other skin types, referred to as the skin type factor (STF), is used to adjust predictions for each skin type: 3.2/3 for skin type II, 3.2/4 for type III, 3.2/5.25 for skin type IV, and 3.2/7.5 for skin type V. We did not calculate vitamin D values for skin type VI because they are below those of skin type V, which has very low values.

The amount of vitamin D_3_ people make from outdoor UV exposures also depends on how much skin they expose to the sun, or the percent body exposure (PBE). To get the best estimates of how much body area people expose or PBE during each season of the year, we used estimates for burn areas (Lund and Browder 1944). For example, we assumed that young adults would expose the face (4.5–7.8%), the front half of the neck (1%), and the front and back of both hands (5%) during all seasons of the year (PBE ~ 10.5–13.8%; Table 1) and would also expose the lower arms (6%) and lower legs (10–13%) during the spring and fall (PBE ~ 30%) and half of the upper arms (4%; short-sleeved/T-shirts) and half of the upper legs (7–9%; shorts/skirts) during the summer (PBE ~ 41–44%).

**Table 1:** Estimates of PBE for each season, by age (based on data from Lund and Browder 1944).

Age (years)						
Exposure (%)	0–5	10	15	≥ 22
Half of head (face)		7.8		5.5		4.5		3.5
Half of neck (front)		1		1		1		1
Hands (front and back)		5		5		5		5
Lower arms		6		6		6		6
Lower legs		10		12		13		14
Half of upper arms		4		4		4		4
Half of upper legs		7		8.5		9		9.5
Total*a*								
Winter		13.8		11.5		10.5		9.5
Spring/fall		30		29.5		29.5		15.5
Summer		40.8		42		43.5		33.5
For summer only								
Upper arms		8		8		8		8
Upper legs		14		17		18		19
Trunk		26		26		26		26
Feet		7		7		7		7
Bathing suit/diaper		85.6		88.5		89.5		90.5
**a**The data for percent body part for the age range of 0–5 years was weighted by age (Lund and Browder 1944).								

Because vitamin D_3_ production decreases with age (MacLaughlin and Holick 1985), we included an age factor (AF) in the calculations. Young adults (< 22 years of age), who have the highest ability to make vitamin D_3_, are assigned an AF = 1, and adults (≥ 22 years of age) are assigned fractions according to their age range (Godar et al. 2011). Therefore, the final equation for calculating the amount of vitamin D_3_ produced from an average everyday UV exposure during each season of the year is


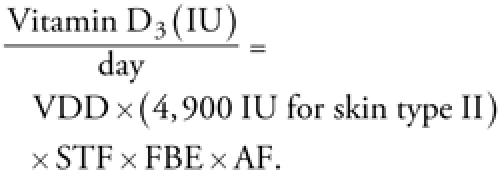
[3]

For example, during the summer, a 4-year-old African-American girl with skin type V living near Atlanta, Georgia (~ 34°N), and wearing a T-shirt and short shorts (thus exposing ~ 40.8% of her body area; see Table 1) would get, on average, about 1 SED/day (Godar 2001) (~ 2 hr outdoors, scattered throughout the day) and would make about 397 IU of vitamin D_3_ per day [vitamin D_3_ (IU/day) = ([1.01 SED × 1.104] × 0.417) × (4,900 IU) × (3.2/7.5) × 0.408 × 1.0].

To account for sunscreen use, divide the estimate from Equation 3 by the SPF factor (Matsuoka et al. 1988). However, this assumes that sunscreens are used correctly, that is, that they are applied generously to the entire body before going outdoors and are reapplied every 2 hr.

To account for additional sun exposure during vacations, we recalculated estimates assuming that 2–3 weeks each summer were spent vacationing at approximately 40°N, as estimated by Godar et al. (2001), with the use of SPF 4 sunscreen or the equivalent only during beach vacations. In addition, we averaged exposures for four different types of vacations—beach, sightseeing, country, and home—and assumed that people would wear the least amount of clothing during a summer vacation (PBE ~ 50–90%), depending on the type of vacation.

## Results

Table 1 shows estimates for PBE by different age groups during each season of the year, based on the data of Lund and Browder (1944). During the winter body exposure is highest in young children (≤ 5 years), and during the rest of the year body exposure is highest in teenagers (13–19 years). Adults (≥ 22 years) have the lowest PBE during all seasons. Note here that levels of vitamin D_3_ can increase during the summer by ≥ 30% (~ 500 IU/day) depending on clothing choice (data not shown).

**Figure 1:**
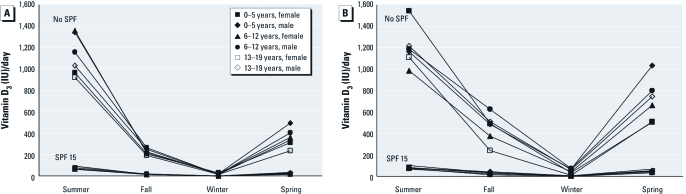
Average estimated vitamin D_3_ production by American children with skin type II according to age (years), sex, season, sunscreen use, and residence in the northern (45°N; *A*) or southern (35°N; *B*) United States.

Figure 1 shows estimates of the average amount of vitamin D_3_ made by children with Fitzpatrick skin type II according to season, age group, sex, diligent use of SPF 15 sunscreen, and residence in the northern or southern United States. According to our estimates, the minimum recommended daily dose of vitamin D (600 IU/day; Institute of Medicine 2011) is achieved by skin type II children in the northern United States only during the summer and only if they do not wear sunscreen with SPF ≥ 15 (Figure 1A). However, results suggest that most children with skin type II in the southern United States can get the recommended daily dose during the spring and summer if they do not wear sunscreen with SPF ≥ 15 (Figure 1B). Optimal vitamin D_3_ production (≥ 1,200 IU/day) is achieved primarily by a subset of children in the southern United States during the summer (Dong et al. 2010).

**Figure 2:**
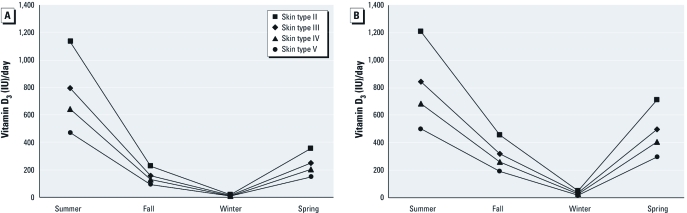
Average estimated vitamin D_3_ produced in children (≤ 19 years of age) from everyday outdoor UV exposures in the northern (45°N; *A*) or southern (35°N; *B*) United States, according to Fitzpatrick skin type and season (without use of sunscreen with SPF ≥ 15).

Figure 2 shows the average amount of vitamin D_3_ produced from everyday outdoor exposure according to skin type (II–V), season, and residence in the northern or southern United States for all children ≤ 19 years combined (without using sunscreen). Our estimates suggest that in the northern United States (Figure 2A), the minimum recommended daily dose of vitamin D_3_ (600 IU/day) is made by skin type II, III, and IV children only during the summer, and the optimal dose of ≥ 1,200 IU/day is not made during any season, regardless of skin type, except by a small subset of skin type II children during the summer (Figure 1A). In the southern United States (Figure 2B), we estimate that the minimum amount (600 IU/day) is achieved during the summer by children with skin types II, III, and IV and during the spring by children with skin type II only, whereas the optimum amount (≥ 1,200 IU/day) is achieved only by children with skin type II during the summer (Figure 2B). These findings suggest that everyday outdoor exposure for children with skin type III and skin type IV rarely provides their minimum vitamin D_3_ needs (~ 600 IU/day), and children with skin type V may never meet their minimum daily vitamin D needs.

**Figure 3:**
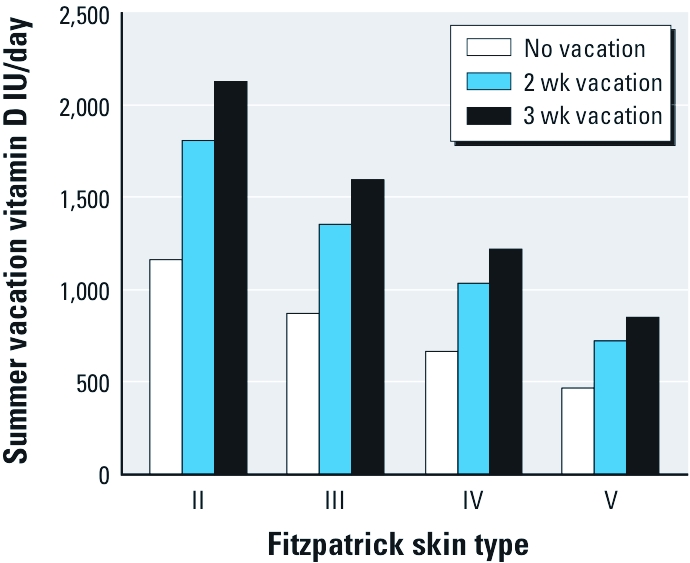
Average estimated vitamin D_3_ made by U.S. children (≤ 19 years of age) during the summer according to skin type and vacation length (at latitude ~ 40°N in the continental United States). We assumed people use the equivalent of SPF 4 sunscreen during beach vacations.

Taking a 2- or 3-week summer vacation at 40°N during the summer increases the average estimated vitamin D_3_ production for children of all skin types but still may be insufficient to meet minimum or optimum requirements in children with skin type V because these are optimistic estimates (minimum clothing and maximum time outdoors and no sunscreens) [Figure 3; UV doses estimated by Godar et al. (2001)].

## Discussion

Although our estimates assumed “optimistic” clothing scenarios for making vitamin D_3_, they raise the question as to whether American children are going outdoors enough to meet their minimum daily vitamin D requirements (600 IU/day; for blood levels of 20 ng/mL or 50 nmol/L) recommended by the Institute of Medicine (2011). The amount of time varies by season (Godar 2001). Based on the results of this analysis, it appears that most active Caucasian children of skin type II can make optimal amounts of vitamin D_3_ (≥ 1,200 IU/day for blood levels of 30 ng/mL or 75 nmol/L) during the summer, but only if they do not use sunscreens diligently except during beach vacations. However, children with darker skin may never achieve optimal production, whereas those with skin types III and IV may meet only minimum requirements during the summer, and children with very dark skin (type V) may meet their optimum requirements during the summer only if they take a 3-week beach vacation at latitudes lower than 40°N (approximate middle of the United States).

If children really need to achieve optimal blood levels of 30 ng/mL (~ 75 nmol/L), then they need at least twice the current recommendation, or about 1,200 IU/day of vitamin D. However, two recent studies, which examined > 25 children in each different skin type and sun exposure category, concluded that children need ≥ 2,000 IU/day from oral supplements (Maalouf et al. 2008), equivalent UV exposure, or a combination (Hall et al. 2010) to maintain blood levels of 25(OH)D > 75 nmol/L. If children really need ≥ 2,000 IU/day, then almost no one in the United States can make the needed amount of vitamin D_3_ from everyday sun exposure all year.

Overall, males go outside somewhat more than females, and children in the youngest age group (≤ 5 years) go outside somewhat more than do those in the other age groups (Godar 2001), so they make a little more vitamin D_3_, in agreement with the findings of three recent large U.S. studies (Ginde et al. 2009; Looker et al. 2008; Mansbach et al. 2009). Children spend approximately 1.6 ± 0.1 hr/day (weekdays and weekends averaged) outdoors during the summer (Godar 2001), giving them about two-thirds of an MED for a skin type II body exposure (Pope and Godar 2010). If the children are Caucasian with skin type II and wear minimal clothing (e.g., wear a diaper, shorts, or a bathing suit, or females wear tank or halter tops and short shorts, or males do not wear a shirt and wear short shorts), then they can make at least 30% more than the estimated vitamin D_3_ (~ 500 IU/day; data not shown). In contrast, if people wear sunscreen with SPF ≥ 15, they will make virtually no vitamin D_3_ (Holick et al. 1995a; Matsuoka et al. 1988).

Our estimates are consistent with reports of vitamin D deficiency (< 50 nmol/L, < 20 ng/mL) and insufficiency (< 75 nmol/L, < 30 ng/mL) among children and adolescents in the United States. For example, the Third National Health and Nutrition Examination Survey (NHANES III 1988–1994) study showed that 13% of male and 29% of female adolescents (12–19 years) had deficient levels and that 25% of male and 47% of female adolescents had insufficient levels of 25(OH)D during the winter (Looker et al 2002). During the summer, 8% of males and 13% of females had deficient levels and 21% of males and 28% of females had insufficient levels of 25(OH)D. In addition, the percentage of children with deficient levels during the winter increased with increasing skin color: skin types I/II, 8% male and 15% female; skin types III/IV, 18% male and 41% female; skin types V/VI, 53% male and 70% female. NHANES 2000–2004 data indicated that approximately 20% more Americans were vitamin D deficient compared with the previous decade, partly because of increased sun protection (Ginde et al. 2009; Looker et al. 2008; Mansbach et al. 2009). A study in Augusta, Georgia (33°N), examined 559 adolescents and found that during the winter, 3.9% of male and 2.6% of female adolescents with skin type II had deficient levels of 25(OH)D, and 46.9% of male and 73.8% of female adolescents had insufficient levels (Dong et al. 2010). The same study also showed that during the winter, 84% of African Americans with skin types V and VI had deficient levels of 25(OH)D and 98% had insufficient levels, whereas during the summer 56% had deficient and 88% had insufficient levels. A study in Pittsburgh, Pennsylvania (40°N), of 41 preadolescent African Americans (6–10 years of age) found that 49% had deficient 25(OH)D levels (Rajakumar et al. 2005). The recent resurgence of rickets in breast-fed African-American infants in several southern states suggests that vitamin D deficiency is on the rise in the United States (Kreiter et al. 2000). Similar insufficient and deficient levels of 25(OH)D are found in young adults in the southern hemisphere at comparable latitudes (35–46°S; Rockell et al. 2005), where they get UV doses similar to those in the United States (Godar 2005). Insufficient sun exposure in adults is also a concern, particularly because the ability to make vitamin D decreases with age; seniors > 70 years of age can make only 25–50% of what a child can make (MacLaughlin and Holick 1985). Deficient and insufficient vitamin D levels from insufficient sun exposure are a worldwide problem with serious health consequences (Holick and Chen 2008).

Conversely, serious health consequences can also arise from too much sun exposure. Too much UV radiation can lead to the formation of three types of skin cancers: squamous cell carcinoma, basal cell carcinoma, and melanoma. Paradoxically, regular, moderate sun exposure may reduce the incidence of fatal melanoma (Godar et al. 2009). Melanoma cells have been shown to convert vitamin D_3_ to 1,25(OH)_2_D *in vitro* (Reichrath et al. 2007), and it has been reported that 1,25(OH)_2_D can reduce tumor growth and decrease the number and size of skin tumors and melanoma xenografts in animal models (Eisman et al. 1987) and inhibit *in vivo* pulmonary metastasis (Yudoh et al. 1999) and angiogenesis (Mantell et al. 2000). Further, continual rather than intermittent outdoor UV exposure has been associated with a reduced incidence of melanoma (Gandini et al. 2005; Kennedy et al. 2003) relative to the cumulative annual UV dose (Godar 2005), and sun exposure has been associated with increased survival in melanoma patients (Berwick et al. 2005). Thus, it has been suggested that promoting protection from all midday UV exposures and advising the diligent use of sunscreens with SPF ≥ 15 may paradoxically be promoting the incidence of melanoma (Godar et al. 2009; Gorham et al. 2007).

In conclusion, our estimates suggest that many children may not get enough sun exposure to meet their minimum daily vitamin D requirements. However, additional research is needed to confirm our estimates and to improve our understanding of the net benefits and risks of sun exposure to children’s health.
